# Culture of Hoffa fat pad mesenchymal stem/stromal cells on microcarrier suspension in vertical wheel bioreactor for extracellular vesicle production

**DOI:** 10.1186/s13287-024-03681-9

**Published:** 2024-03-05

**Authors:** Alexander Otahal, Karina Kramer, Markus Neubauer, Slavomira Gulová, Zsombor Lacza, Stefan Nehrer, Andrea De Luna

**Affiliations:** 1 Center for Regenerative Medicine, Department for Health Sciences, Medicine and Research, University for Continuing Education Krems, Krems, Austria; 2 Department of Orthopaedics and Traumatology, Universitätsklinikum Krems, Krems, Austria; 3 Department of Sport Physiology, University of Physical Education, Budapest, Hungary; 4 Inst. Clinical Experimental Research, Semmelweis University, Budapest, Hungary; 5 Associated Tissue Bank, Faculty of Medicine, Pavel Jozef Safarik University and Louis Pasteur University Hospital, Kosice, Slovakia

**Keywords:** Mesenchymal stem cell, Extracellular vesicle, Bioreactor, 3D-culture, Cartilage, Hoffa fat pad, Infrapatellar fat pad, Differentiation, Adipose stem cells

## Abstract

**Background:**

Mesenchymal stromal cells (MSCs) are increasingly employed in regenerative medicine approaches for their immunomodulatory and anti-inflammatory properties, which are encoded in their secretome including extracellular vesicles (EVs). The Hoffa fat pad (HFP) located infrapatellarly harbours MSCs that could assist in tissue homeostasis in osteoarthritic joints. Intraarticular injection therapies based on blood products could modulate the populations of released HFP-MSC-EVs in a quantitative manner.

**Methods:**

To obtain amounts of HFP-MSC-derived EVs that allow pre-clinical evaluation, suitable EV production systems need to be developed. This work investigates the release of EVs from primary HFP-MSCs cultivated in a 3D environment using microcarrier suspension culture in a vertical wheel bioreactor in comparison to conventional 2D culture. To simulate an intraarticular blood product therapy, cultures were treated with citrate-anticoagulated platelet-rich plasma (CPRP) or hyperacute serum (hypACT) before EV collection. HFP-MSC-EVs are enriched via ultrafiltration and characterised via Western Blot, nanoparticle tracking analysis in scatter as well as fluorescence mode. EV potency was determined via RT-qPCR analysing the expression of type II and X collagen (COL2 and COL10), as well as inducible nitric oxide synthase (iNOS) in primary OA chondrocytes.

**Results:**

Blood product supplementation elevated HFP-MSC metabolic activity as determined via XTT assay over the course of 14 days. 3D culture resulted in a roughly 100-fold EV yield compared to 2D culture and elevated number of EVs released per cell. Total protein content correlated with the EV concentration. While typical EV marker proteins such as CD9, CD63 or Alix were detected in total protein extracts, CD9 and CD73 colocalised on individual EVs highlighting their cell origin. The type of blood product treatment did not affect the size or concentration of EVs obtained from HFP-MSCs. Assessing potency of 3D culture EVs in comparison to 2D EVs revealed superior biological activity with regard to inhibition of inflammation, inhibition of chondrocyte hypertrophy and induction of cartilage-specific ECM production.

**Conclusions:**

HFP-MSCs proliferate in presence of human blood products indicating that animal serum in culture media can be avoided in the future. The culture of HFP-MSCs in the employed bioreactor was successfully used to generate quantities of EVs that could allow evaluation of HFP-MSC-EV-mediated effects in pre-clinical settings. In addition, EV potency of 3D EVs is superior to EVs obtained in conventional 2D culture flasks.

**Supplementary Information:**

The online version contains supplementary material available at 10.1186/s13287-024-03681-9.

## Background

The Hoffa fat pad (HFP), also known as the infrapatellar fat pad, is a specialized fat tissue located in the anterior part of the knee joint. It is anatomically positioned intra-articularly, but extra-synovially. It is an important anatomical part of the knee joint and has several functions. The HFP plays an important role in the lubrication and cushioning of the knee joint. It helps to reduce friction between the patellar tendon and the underlying bones and cartilage as well as to distribute load and pressure throughout the joint during movement (Ioan-Facsinay et al., 2013). Like other types of fat tissue, the HFP contains multipotent mesenchymal stromal cells (MSCs) [40]. MSCs from various sources are explored in numerous cell therapeutic approaches in regenerative medicine including osteoarthritis (OA) [21, 29, 30, 34], however, there is mounting evidence that factors contained in the secretome of MSCs are the main drivers of therapeutic outcomes [24, 37]. In particular, extracellular vesicles (EVs) are responsible for regenerative effects mediated by MSCs in the context of OA observed in several studies [5, 38, 42]. The vicinity of the HFP to affected regions in OA led to the hypothesis that MSCs from this tissue and their secretome including EVs could be involved in tissue homeostasis of the knee joint and could consequently be employed in a regenerative treatment approach for OA.

Our initial experiments on producing HFP-MSC-EVs in conventional 2D culture flasks yielded only around 10^8^ EVs/ml conditioned medium. This concentration was too low for the implementation of a rational EV production procedure to yield EVs in quantities allowing pre-clinical evaluation. Three-dimensional culture systems exert mechanical stimulation on cells. This favoured not only increased EV release from MSCs [6] or in vivo-like characteristics of EVs in terms of their cargo [36], but also elevated their bioactivity in a therapeutic context [41]. Therefore, a vertical wheel bioreactor (VWBR)-based approach [33] was implemented involving culture of HFP-MSCs on microcarriers in suspension to circumvent the observed bottelneck of low EV yield.

An increasingly applied procedure in orthopaedic care to treat OA is intraarticular injection of blood derived products such as platelet-rich plasma (PRP). There is evidence that these treatments result in beneficial and diseases modifying outcomes [4, 11]. HFP-MSCs might respond to these treatments via modulation of their secretome including EV populations. To mimic such treatments in the employed bioreactors, the cultures were treated with citrate-anticoagulated platelet-rich plasma (CPRP) or hyperacute serum (hypACT) and resulting EV populations were compared quantitatively to EVs obtained from HFP-MSCs grown in conventional FCS-supplemented media.

Therefore, to establish an EV production protocol from primary OA HFP-MSCs the aims of this study include (1) implementing a culture protocol for patient-derived HFP-MSCs on microcarriers in a vertical wheel bioreactor (VWBR) for EV production, (2) implementing an enrichment procedure of HFP-MSC-derived EVs from the bioreactor culture via ultrafiltration, (3) characterising HFP-MSC-derived EVs qualitatively and in terms of quantity in response to blood product treatment compared to conventional 2D culture, and (4) comparing the potency of HFP-MSC-EVs from VWBR culture and conventional 2D culture on primary OA chondrocytes.

## Methods

### Tissue harvest and cell isolation

HFP tissue was obtained from 4 osteoarthritic patients undergoing total joint replacement surgery and 1 patient undergoing knee arthroscopy after obtaining informed consent under approval by the ethics committee from Karl Landsteiner Private University (EK 1020/2020). Cell isolation was performed as previously described [20, 25]. Briefly, HFP tissue was minced into 5 mm pieces with a scalpel and digested with 9000 units of collagenase I (Sigma-Aldrich, #C0130) per 10 g of HFP in 15 ml DMEM (GIBCO, #21969-035) for 2 h at 37 °C in a 50 ml tube on a roller. Afterwards, the suspension was filtered through a cell strainer (Corning, #431,750), cells were washed once in PBS and resuspended in growth medium containing DMEM, high glucose, GlutaMAX™ and pyruvate (GIBCO, #31,966,047), 2% Penicillin/Streptomycin (Sigma-Aldrich, #P4333), 1% Amphotericin B (Sigma-Aldrich, #A2942), 10% heat inactivated fetal calf serum (GIBCO, #11,550,356 ), 1% nonessential amino acids (GIBCO, #11,140,050) and 1 ng/ml bFGF (Sigma-Aldrich, #F0291). Cells were seeded at a density of 10,000 cells/cm2 in T175 flasks for expansion culture at 37 °C/5% CO_2_. Human articular cartilage was obtained from one male patient undergoing joint replacement surgery at the Universitätsklinikum Krems. Informed consent was obtained from the patient. Chondrocytes were isolated and cultured as previously described (De Luna-Preitschopf et al., 0.2017).

### Preparation of blood products CPRP and hypACT

Whole blood was collected in-house from 5 to 7 donors chosen randomly from a pool of volunteers after an informed consent was signed. Blood collection was approved by the local Ethics Committee of the Danube University Krems (ESC1020/2020). Inclusion criteria for blood donation were age between 25 and 45 as well as being healthy on the day of blood donation according to an evaluation form asking for conditions such as pregnancy, underweight or diabetes as exclusion criteria. Citrate anticoagulated PRP (CPRP) was generated via collecting whole blood into citrate-coated vacutainer tubes (VACUETTE 9NC trisodium citrate 3.2%, Greiner BioOne, #455,322) and further processed as previously described [20]. hypACT was prepared via reconstitution of freeze-dried hypACT serum powder (kindly provided by Orthosera GmbH) with 2 ml sterile double distilled water.

### Metabolic activity via XTT assay

To determine metabolic activity after cell isolation, 1000 cells per well were seeded in 96 well plates in triplicates, with separate wells for each time point up to 14 days. To monitor metabolic activity in the VWBR, an aliquot of 250 µl was drawn from the cell suspension and 20 µl suspension was added to 80 µl growth medium per well in 96-well plates in triplicates. After adding 50 µl XTT reagent prepared according to the manufacturer’s recommendations (Roche, #11,465,015,001) per well and incubation for 4 h at 37 °C / 5% CO_2_, absorbance was measured at 492 nm with 690 nm correction on a Synergy 2 plate reader. A blank was prepared for each measurement by adding XTT reagent to medium only.

### Differentiation of isolated MSCs

Tri-lineage differentiation was performed for 5 donors. Cells were cultured in growth medium or differentiation media as described below supplemented with either 10% CPRP, 10% hypACT or 10% FCS as control. As CPRP required addition of 2 U/ml heparin (Gilvasan Pharma GmbH, #3,909,969) into the culture medium, all conditions were performed in presence of heparin.

#### Adipogenic differentiation

The adipogenic differentiation potential was determined via Oil red O staining as previously described [20]. In brief, HFP-MSCs were seeded into 6 well plates at a density of 2*10^5^ cells and were cultured in normal growth medium until 100% confluency for 3–5 days, before differentiation was started. Differentiation medium was prepared from serum free growth medium containing StemXVivo^®^ Adipogenic Supplement (Bio-Techne Ltd., Abingdon, United Kingdom). It was further supplemented with either 10% CPRP, 10% hypACT or 10% FCS as control and was changed every 3–4 days. After 21 days, cells were rinsed with PBS, fixed in 10% formalin for 30 min and stained with Oil Red O (Sigma, #01391) according to the manufacturer’s recommendations. Fixed cells were washed with 2 ml distilled water, before 1 ml 60% isopropanol was added for 2 min. Afterwards, 1 ml Oil Red O working reagent (3 parts Oil Red O stock solution, 2 parts distilled water) was added for 5 min. Then, excess dye was gently washed away with distilled water until the water stayed clear. Stained cells were visualised using a phase contrast microscope. Additionally, incorporated dye was extracted via 300 µl 100% isopropanol, centrifuged at 14,000 x g for 5 min and absorbance was measured for 80 µl supernatant in triplicates at 490 nm on a Synergy 2 plate reader.

#### Chondrogenic differentiation

The chondrogenic differentiation potential was determined via chondropellet formation as previously described [7]. Briefly, 2.5*10^5 cells were mixed with chondrogenic medium composed of DMEM high glucose (GIBCO, #10,596,010), 1% insulin-transferrin-selenium (ITS) liquid media supplement (Sigma-Aldrich, #I3146), 100 nM dexamethasone (Sigma-Aldrich, #D4902), 50 µg/ml ascorbic acid (Sigma.Aldrich, #A4403), 1% non-essential amino acids (GIBCO, #11,140,050), 5 ng/ml TGFβ-3 (PeproTech, #AF-100-36E), and 4% methylcellulose (Sigma-Aldrich, #M7027) and were centrifuged at 4164 x g for 10 min. Pellets were cultured for 21 days at 37 °C/5% CO_2_ in 15 ml tubes with loose cap and media were changed twice a week. Then, pellets were recovered from the culture medium and excess liquid was removed. Pellets were put on base molds (Fisher Scientific, #22-363-554) prepared with a layer of frozen tissue matrix (Tissue-Tek® O.C.T.™, Sakura Finetek, #4583) and put at -80 °C for at least 24 h. Cryosectioning was performed on a cryostat device (Cryostar NX70, Thermo Fisher Scientific) and 6 μm thick slices were placed on adhesive glass slides (Thermo Scientific™ SuperFrost Plus™, Thermo Fisher Scientific, #10,149,870). Slides were dried and fixed in cold acetone (-20 °C) for 10 min. Histologic staining was performed with Alcian Blue 8GX (Sigma-Aldrich, #A3157) prepared from 1 g Alcian Blue 8GX, 97 ml distilled water and 3 ml 96% acetic acid (Sigma-Aldrich, #A9967) for 30 min. Sections were washed in running tap water for 1 min, dehydrated through 95% ethanol and 2 changes of absolute ethanol for 3 min each. Slides were mounted with xylene before adding a cover glass. Sulfated glycosaminoglycans appeared blue and were visualised in a light microscope to determine the diameter of the chondropellets.

#### Osteogenic differentiation

The osteogenic differentiation potential was determined via Alizarin S staining as previously described [25]. In brief, HFP-MSCs were seeded into 6 well plates at a density of 2*10^5 cells and were cultured in normal growth medium until 100% confluency for 3–5 days, before differentiation was started. Differentiation medium (serum free growth medium supplemented with 100 nM dexamethasone (Sigma-Aldrich, #D4902), 50 µg/ml ascorbic acid (Sigma-Aldrich, #A4403) and 10mM β-glycerol phosphate (Sigma-Aldrich, #G9422)) supplemented with either 10% CPRP, 10% hypACT or 10% FCS as control were changed twice a week. After 21 days, cells were rinsed with PBS, fixed in 10% formalin for 30 min and stained with aqueous 2% Alizarin Red S solution (Sigma-Aldrich, # A5533), pH 4.3, via covering the cells with 1 ml dye solution for 30 min at room temperature. Afterwards, cells were rinsed with distilled water 3 times and mineralized areas appearing red were visualized via light microscopy. Then, stained cells were incubated with 500 µl 10% acetic acid for 30 min, collected with a cell scraper, vortexed and heated at 85 °C for 10 min, cooled down, and centrifuged at 20,000×g for 15 min. The supernatant was neutralized with 500 µl 10% ammonium hydroxide and absorbance was measured for 100 µl in triplicates at 405 nm with a Synergy 2 plate reader.

### Flow cytometry

HFP-MSCs adhering to cell culture dish 48 h post isolation or after EV collection 48 h after switching the cultures to serum free medium were used for flow cytometry employing the StemFlow kit (BD, #562,245). 10^5^ cells were stained with 4 µl positive or negative antibody cocktail and 1 µl compensation control antibodies were used. The positive markers (CD90, CD105, CD73 and CD44) and negative markers (CD34, CD11b, CD19, CD45, HLA-DR) were involved in the analysis. Data acquisition and analysis was performed with a CytoFlex device and FlowJo software vX.0.7, respectively.

### EV production

**Fig. 1:**
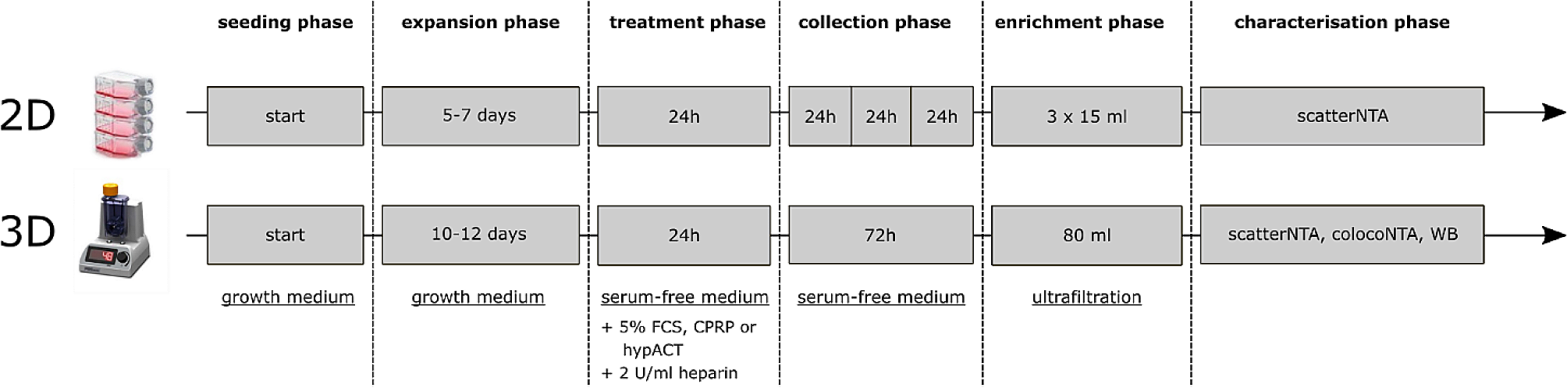
Schematic overview of 2D and 3D HFP-MSC-EV production protocols. For 2D EVs characterisation, only particle concentration was assessed via NTA in scatter mode. The observed low EV yield led to the establishement of a 3D EV production procedure using vertical wheel bioreactors

HFP-MSCs were seeded into either standard cell culture flasks or into vertical bioreactor culture chambers (PBSMini, PBSBiotech) after detachment from 90% confluent 2D cultures via accutase (GIBCO, #A1110501) and counting via trypan blue dye exclusion using a Neubauer counting slide. For 2D and 3D EV production protocols as described below and outlined in Fig. 1, HFP-MSCs were used having a passage number lower than 5 at seeding for EV production.

### 2D protocol

Cells were seeded at 10,000 per cm^2^ in T175 flasks in 30 ml growth medium and cultured until 80–90% confluency with growth medium change twice per week. The expansion phase usually lasted 7 days. Afterwards, medium was switched to serum free medium supplemented with 5% (v/v) of either FCS, CPRP or hypACT and 2 U/ml heparin (Gilvasan, #3,909,981) to mimic an intraarticular blood product treatment for 24 h. Then, medium was switched to 15 ml serum free medium to start EV collection phase. Conditioned media were harvested daily for 3 days and replaced with 15 ml fresh serum free medium. Harvested media were pooled per condition and donor and stored at -80 °C until further processing.

### 3D protocol

On average, 4.33*10^6^ cells were mixed with 1.6 g Synthemax II microcarrier beads (Corning, #3781). The beads have a surface of 360 cm^2^/g, therefore the seed density corresponded to around 7,500 cells/cm^2^. Cells and microcarriers were suspended in 25 ml growth medium and put on a roller for 1 h at 37 °C with 5 rpm, with 2 min agitation phases and 5 min settling phases to allow for cell attachment. Then, the suspension was transferred to the PBSMini vertical wheel bioreactor chamber (PBSBiotech, USA) with a 25 ml pipette and 50 ml growth medium was added. Rotator speed was set to just keep the beads suspended. With increasing culture time, the wheel speed was increased from 18 to 25 rpm. During expansion phase, 20 ml growth medium was changed daily. Afterwards, medium was switched to serum free medium supplemented with 5% (v/v) of either FCS, CPRP or hypACT and 2 U/ml heparin (Gilvasan, #3,909,981) to mimic an intraarticular blood product treatment for 24 h. Then, the cell-bead suspension was transferred to 50 ml tubes for washing once with PBS via centrifugation at 200 x g for 5 min, before resuspending in serum free medium, returning to the bioreactor chamber and continuing culture in 80 ml total medium volume for 72 h for EV collection. Afterwards, conditioned supernatant was harvested via gravitational settling of the cell-microcarrier suspension and transfer of the clear supernatant to 50 ml tubes. After centrifugation at 4,000 x g for 15 min to remove residual cells and cell debris, the cleared conditioned supernatant was stored at -80 °C until further processing.

### Ultrafiltration of conditioned supernatant

Conditioned supernatant stored at -80 °C was thawed at 37 °C in a water bath. Volumes of 45 ml or 80 ml were processed for 2D or 3D cultures, respectively. To prepare the 100 kD Amicon ultrafiltration units (Merck, #UFC9100), one spin with PBS followed by one spin of 70% ethanol, again PBS and one spin of ultrafiltrated 1% BSA in PBS was performed to ensure sterility and to reduce unspecific attachment of EVs to the filter unit during sample processing. Conditioned supernatants were processed in aliquots of 15 ml per centrifugation round (4,000 x g, 15 min, 4 °C) using the same filter unit per sample to accumulate retentates. After the whole conditioned supernatant was filtered and EVs accumulated in the filter unit, 15 ml PBS were added to the filter unit to wash the EVs via resuspending the retentate and centrifuging again at 4,000 x g for 15 min at 4 °C. The primary retentate was then recovered as fraction “EV” via pipetting into 1.5 ml low attachment tubes (Biozym, #710,176). Then, 1 ml PBS was added to the filter unit, which was then vortexed at 2,000 rpm for 20 s to detach remaining EVs from the filter membrane. This cleanout procedure was performed 5 times consecutively to obtain fractions “W1”-“W5” to achieve quantitative recovery of EVs.

### Nanoparticle tracking analysis (NTA)

Size and concentration of particles were determined via NTA in scatter mode on a Particlemetrix MONO device, while colocalisation experiments were performed on a TWIN device. Samples were diluted as required 1:100-1:1000 with PBS. Firstly, scatter mode video acquisition at 11 positions with 30 frames per second for 2 s each used the camera settings sensitivity 80 and shutter 100. Video acquisition and analysis was done with ZetaView 8.04.02 or Zetanavigator 1.0.3.8.6 and ParticleExplorer 4.0.5.4 software. Secondly, for colocalisation of CD9 and CD73 on EV samples, antibodies were 1:10 prediluted in PBS. 8 µl of EV suspension were mixed with 1 µl CD9 1:10 and CD73 1:10 and incubated in the dark for at least 1 h. Afterwards, stained EV suspensions were diluted 1:333 in PBS for measurement at camera sensitivity 95 to determine the percentage of CD9 positive particles colocalising with CD73 and vice versa.

### Induction of senescence

To prepare control protein extracts of HFP-MSCs to rule out senescence induction due to bioreactor culture, HFP-MSCs were seeded at 10,000 per cm^2^ in 6-well plates in growth medium. After 48 h, 5 µM etoposide (Sigma, # e1383) were added for 48 h as previously described [16], or 30 µM H_2_O_2_ (approximately 0.001% (w/v) for 2 h were added similar to a previous study [10]. Afterwards, cells were processed for protein sample preparation as described below.

### Protein quantification

Total protein was determined via BioRad DC assay in microplate format. Ten microlitres of undiluted or diluted sample as required were used. Samples were lysed with 10 µl RIPA buffer without protease inhibitors (Thermo Scientific, #89,900) for 10 min at 4 °C to release EV protein content prior to performing the assay. A linear standard curve ranging from 2.0 to 0 mg/ml was generated with bovine serum albumin (BSA) (Sigma-Aldrich, #A8022). After 15 min incubation in the dark, absorbance at 670 nm was determined using a plate reader.

### Western blot

HFP-MSCs or HFP-MSC EVs were lysed in RIPA buffer (Sigma #R0278) supplemented with phosphatase and protease inhibitor cocktail (Thermo Scientific, #78,440) via vigorous pipetting and incubation at 4 °C for 15 min. Following protein quantification, 10 µg total protein were separated on 10% or 4–12% gradient SDS-PAGE pre-cast gel electrophoresis (Invitrogen, #NP0322). Samples were reduced via 2 µl 1 M dithiothreitol for detection of Alix, ApoB100, p21 and (cleaved) caspase 3, while samples were separated unreduced to probe for CD9 and CD63. After semidry blotting onto PVDF membranes, the following primary antibodies were used 1:1000 diluted in 1% BSA/PBST: Alix (Cell Signaling, #2171), CD63 (BioLegend, #353,005), CD9 (System Biosciences, #EXOAB-CD9A-1), ApoB100 (SantaCruz, #sc-393,636), caspase 3 (Cell Signaling, #14,220), cleaved caspase 3 (Cell Signaling, #9661), p21 (Cell Signaling, #2947), GAPDH (Abcam, #ab37168). Detection was performed via enhanced chemiluminscence (ECL) using WesternBright ECL substrate (Advansta, #K-12,045-D20). Automatic white correction was applied to blot images via GIMP software version 2.10 before semiquantitative analysis was performed densitometrically using the GelAnalyzer plugin of ImageJ software version 1.51s.

### RNA extraction and reverse transcription quantitative PCR (RT-qPCR)

Total RNA was extracted from chondrocytes cultured in the inflammation model and M1 macrophages using a High Pure RNA Isolation Kit (Roche, #11,828,665,001). A Transcriptor cDNA Synth Kit (Roche, #04897030001) was used to synthesize cDNA according to the manufacturer’s protocol. For qPCR, FastStart Essential DNA Probes Master (Roche, #06402682001) was mixed with 1 µL cDNA and 900 nM of primer (Supplementary table 1). CT values were obtained on a LightCycler 96 device (Roche, #05815916001). Data were normalized to GAPDH, and fold changes were calculated via the ΔΔCt method.

### Statistics

Statistical analyses were performed via GraphPad Prism 9.5 and statistical significance was accepted for *p* < 0.05. Data were tested for normality via Shapiro-Wilk test and analysed via one-way ANOVA with Tukey post-hoc test or two-way ANOVA with Sidak post-hoc test as indicated in the figure legends. Correlation analyses were performed via Pearson or Spearman correlation analysis for normally or not normally distributed data, respectively. Data in graphs are represented as mean ± SD, unless otherwise stated.

## Results

### Characterisation of isolated HFP-MSCs

Hoffa fat pad tissue was obtained from 5 donors. The donor details are given in Table 1. On average, 6.48 ± 2.68 (SD) g of Hoffa fat pad tissue was used for cell isolation yielding 2.46*10^6^ ± 1.54*10^6^ (SD) cells per gram tissue.

**Table 1:** Details of the study population. F . female, M . male, BMI . body mass index, n.d. . not determined

Donor	age	gender	weight [kg]	height [cm]	BMI	tissue [g]	cells / g
1	80	F	79	165	29.02	7	2.06*10^6^
2	81	F	110	178	34.72	5	1.92*10^6^
3	46	M	93	182	28.08	3.9	4.69*10^6^
4	82	M	80	178	25.25	10	1.18*10^6^
5	27	M	120	183	35.83	n.d.	n.d.
Average ± SD	63.2 ± 17.52		96.4 ± 14.48	177.2 ± 7.41	30.58 ± 3.97	6.48 ± 2.68	2.46*10^6^ ± 1.54*10^6^

**Fig. 2:**
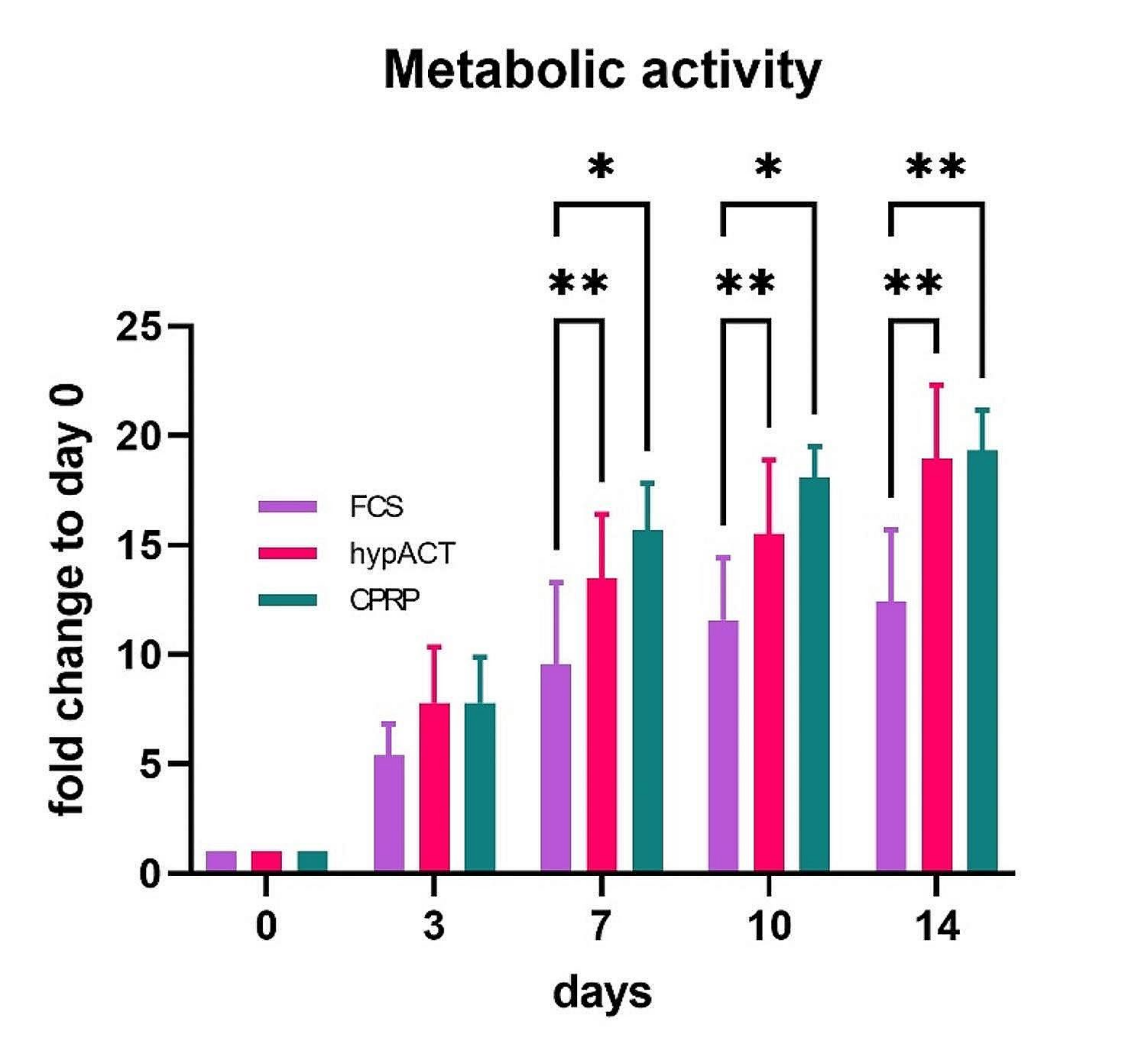
Metabolic activity of isolated cells as determined via XTT assay. Data are shown as arithmetic mean of 4 individual donors ± SD. * . *p* < 0.05, ** . *p* < 0.005

XTT assay was performed to assess the viability of isolated HFP-MSCs (Fig. 2). Over the course of 14 days, metabolic activity increased, and cells cultured in FCS-supplemented media were consistently less metabolically active than hypACT or CPRP supplemented cells from day 7 on (F(1.773,5.319) = 12.89, *p* = 0.001, 2-way ANOVA). CPRP and hypACT supplemented cells had comparable metabolic activity throughout the observation period.

The surface marker expression of isolated cells was assessed via flow cytometry determining the presence of classical MSC markers (CD73, CD90, CD105) and CD44 as well as absence of negative markers (CD34, CD11b, CD19, CD45, HLA-DR) as shown in supplementary Fig. 1. The histograms evidently show that CD73, CD9, CD105 and CD44 were expressed on the isolated HFP-MSCs, while the negative marker cocktail proves the absence of hemapoietic cells, leukocytes or endothelial cells. The mode fluorescence intensity in relation to the respective isotype controls (ΔMFI) for the indicated markers is shown in the bar plot in Fig. 2. The signal intensity for CD105 was substantially lower than for CD90, CD73 or CD44, however, does not necessarily indicate a low abundance of CD105. These results qualify the cell population as MSCs devoid of hemapoietic cells, endothelial cells or leukocytes.

**Fig. 3:**
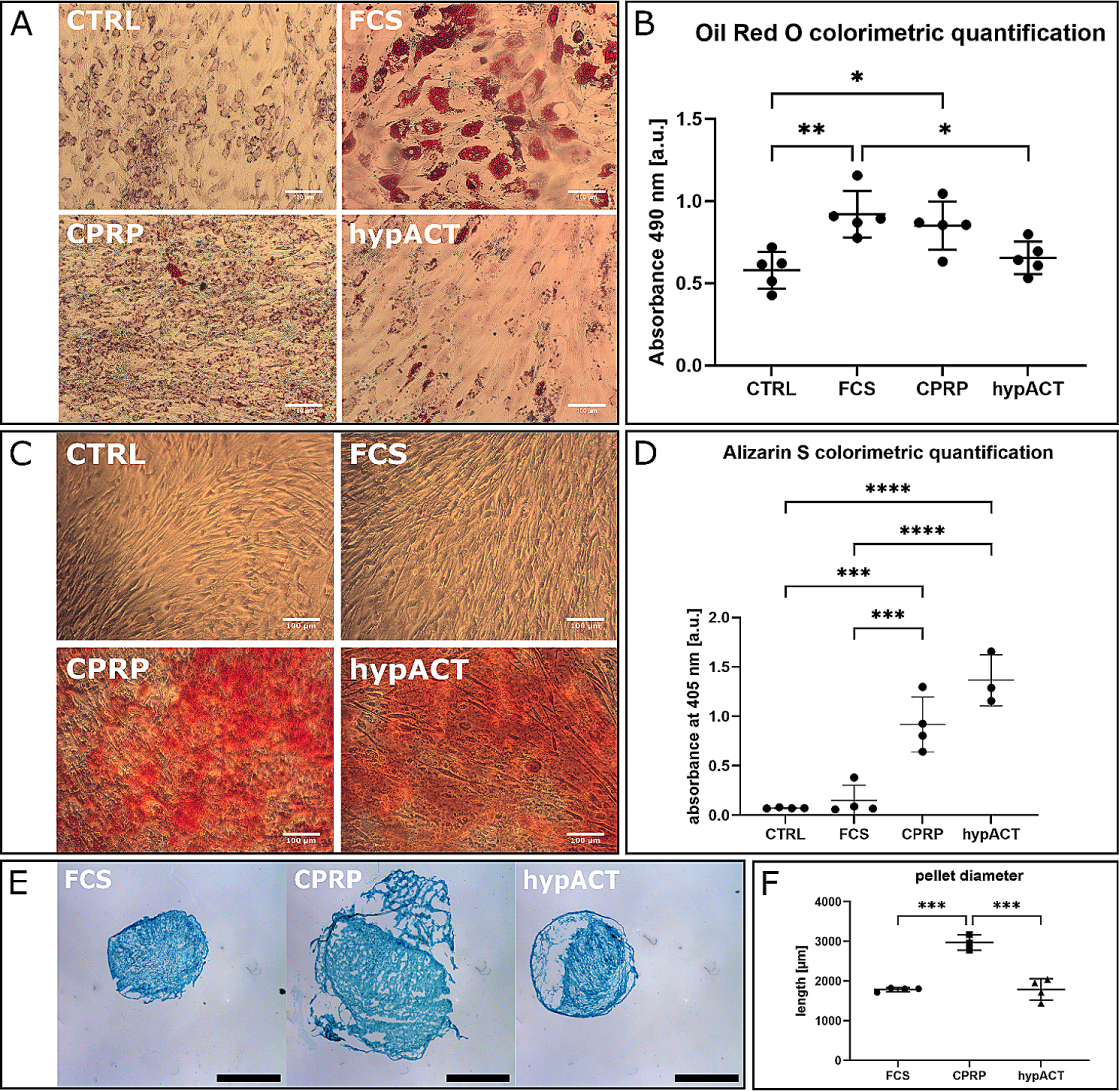
Trilineage differentiation potential of HFP-MSCs. (A) Adipogenic differentiation of HFP-MSCs indicated by lipid droplets stained with Oil Red O from a representative donor. Scale bar 100 μm. (B) Colorimetric quantification of Oil Red O extracted from lipid droplets. (C) Osteogenic differentiation indicated by deposition of calcified material stained with Alizarin S. (D) Colorimetric quantification of Oil Red O extracted from calcified material. (E) Chondropellet formation in presence of differentiation medium supplemented with FCS, CPRP or hypACT. Scale bar 1 mm. Images were uniformly contrast enhanced using automatic white correction via GIMP 2.8.22 software (F) Diameter of chondropellets in presence of FCS, CPRP or hypACT. Data are represented as mean ± SD. *n* = 3–5, * . *p* < 0.05, ** . *p* < 0.005, *** . *p* < 0.001, **** . *p* < 0.0001

To characterise the differentiation capacity of the isolated HFP-MSCs, cells were differentiated in 6-well plates into adipocytes and osteocytes as well as into chondrocytes in 3D pellet cultures. Figure 3A-B illustrates the formation of lipid droplets stained in red in response to adipogenic differentiation medium. Colorimetric quantification of Oil Red O uptake revealed significant differences of HFP-MSCs differentiated in presence of either FCS, CPRP or hypACT in comparison to HFP-MSCs cultured in normal growth medium (F (3, 16) = 8.118; *p* = 0.0016). FCS-supplemented cells retained more lipid dye than either undifferentiated cells (*p* = 0.0029) or hypACT-supplemented cells (*p* = 0.02). CPRP-supplemented cells accumulated more lipid dye than undifferentiated cells (*p* = 0.017), however, the concentration of extracted lipid dye was on average similar to FCS- and hypACT-supplemented differentiated cells.

As shown in Fig. 3C-D, osteogenic differentiation was successful, however, significant differences were observed depending on the media supplementation (F (3, 11) = 34.13; *p* < 0.0001). Deposition of calcified material was strongly promoted by supplementation with CPRP or hypACT compared to undifferentiated cells (*p* = 0.0004 or *p* < 0.0001, respectively) or FCS-supplemented cells (*p* = 0.001 or *p* < 0.0001, respectively). Again, no significant difference between CPRP of hypACT supplementation was observed (*p* = 0,0564). One outlier (0.143) was removed from the hypACT group.

Chondrogenic differentiation was assessed in 3D pellet culture and the size of the chondropellets was measured (Fig. 3E-F). Chondropellet sizes of 1.792 ± 0.049 mm (SD), 2.974 ± 0.188 mm (SD) and 1.791 ± 0.267 mm (SD) were observed for pellets cultured in differentiation media supplemented with FCS, CPRP or hypACT, respectively. According to ANOVA (F(2,8) = 41.67; *p* < 0.0001), presence of CPRP resulted in significantly larger pellets than presence of FCS or hypACT (*p* = 0.0001 or *p* = 0.0001, respectively).

### Bioreactor monitoring

**Fig. 4:**
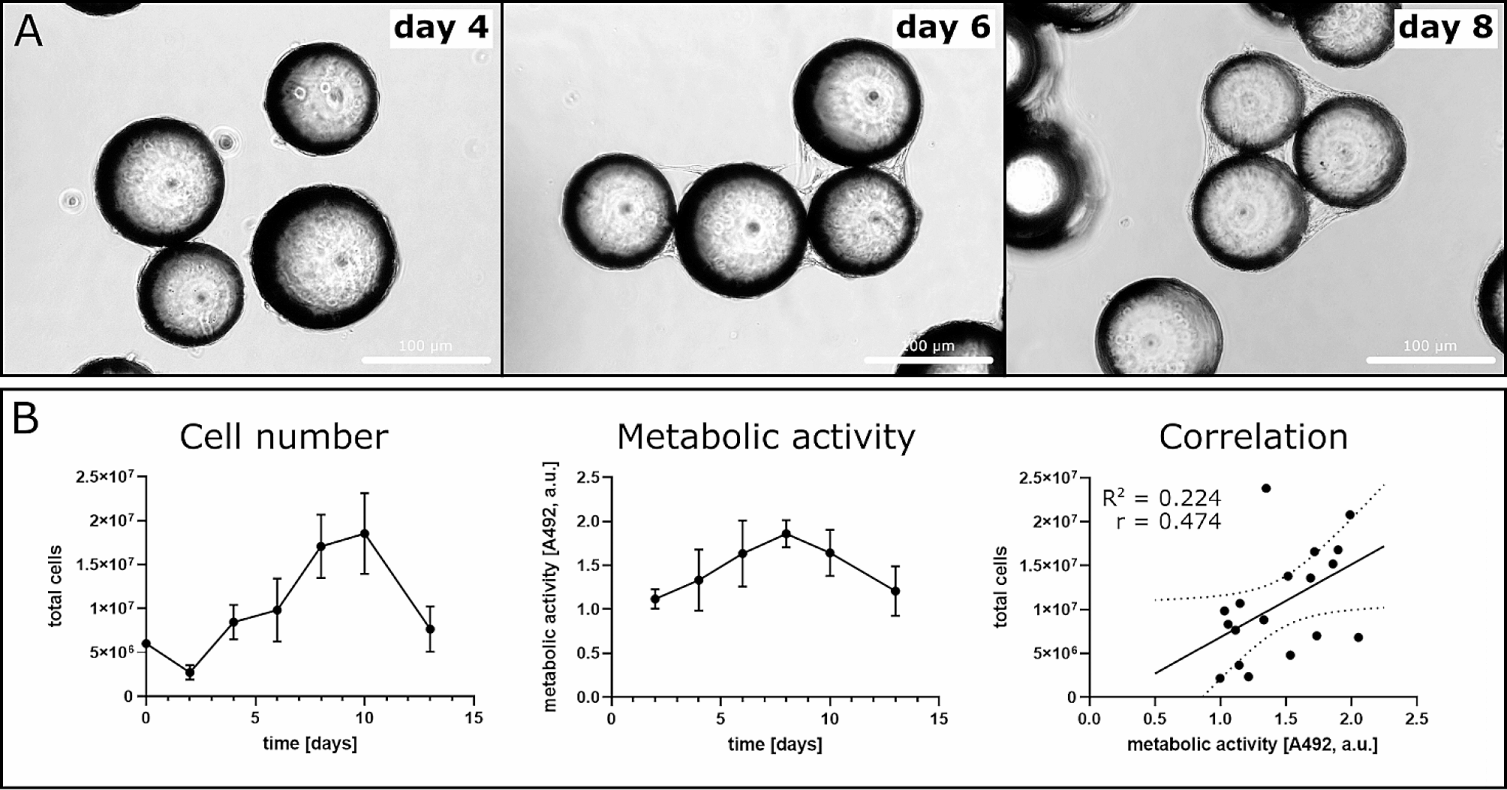
Monitoring of HFP-MSC culture in bioreactor. (A) Micrographs of cell-seeded microcarrier beads on day 4, 6 and 8 showing the formation of cell-bead aggregates/clusters. Scale bar 100 μm. (B) Cell number and metabolic activity over time and their correlation in a representative batch of 3 bioreactors operated in parallel. Data are given as mean ± SD, dotted lines indicate 95% confidence interval, *n* = 3 biological replicates of one representative donor

To monitor the proliferation of HFP-MSCs within the vertical wheel bioreactor and to estimate the ideal time to start EV collection, metabolic activity was regularly determined via XTT assay. The number of cells per volume was determined via counting cells enzymatically detached from beads from an aliquot of the suspension culture via trypan blue dye exclusion. Cell-bead aggregates/clusters started to form on day 4, with increasing cluster size over time (Fig. 4A). Formation of clusters required increasing the rotor speed to 25–30 rpm to prevent settling of the suspension. Cell count and metabolic activity data were normally distributed (*p* = 0.133 and *p* = 0.525, respectively) and correlated positively (*r* = 0.474) with significance (F = 4.629, *p* = 0.047), but with a low goodness of fit of a linear regression curve (R^2^ = 0.224).

**Fig. 5:**
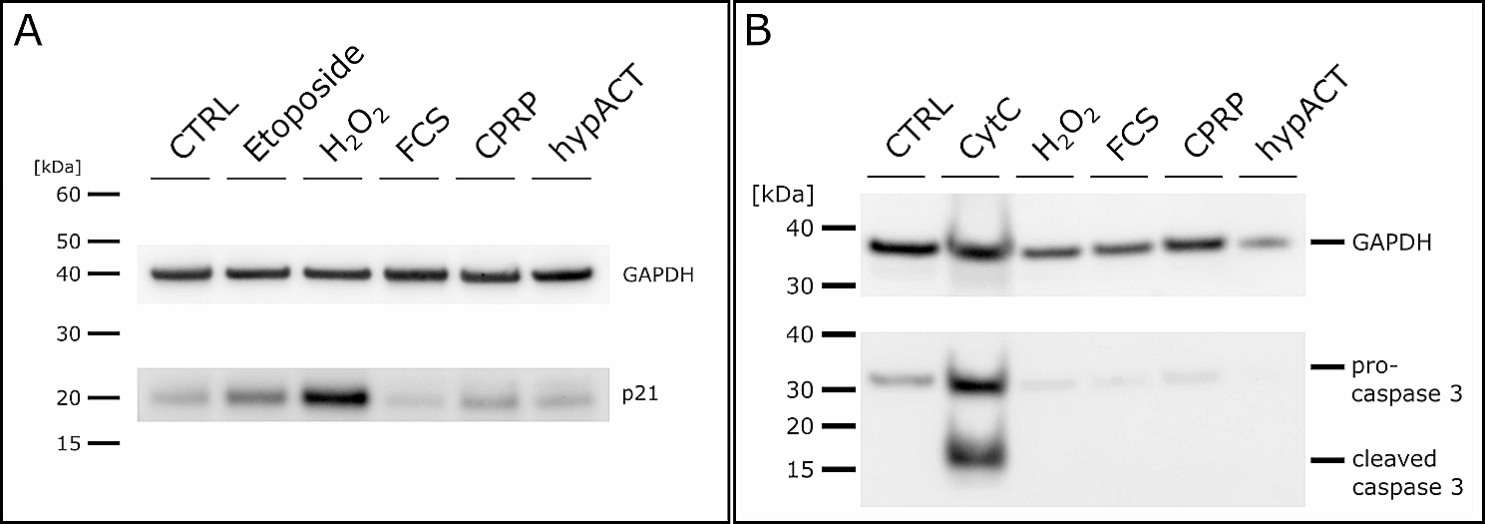
Probing HFP-MSC cell lysates before and after 3D bioreactor culture senescence or apoptosis markers to rule out activation of these cellular processes during EV collection. (A) Analysis of p21 expression before (CTRL) and after EV collection following treatment of HFP-MSCs with the indicated blood product (FCS, CPRP, hypACT). Lysates of HFP-MSCs treated in 2D with 5 µM etoposide or 30 µM H_2_O_2_ for 48 h and 2 h, respectively, were used as positive controls. (B) Analysis of caspase 3 cleavage before (CTRL) and after EV collection following treatment of HFP-MSCs with the indicated blood product (FCS, CPRP, hypACT). Lysates of HFP-MSCs treated in 2D with H_2_O_2_ or cytochrome C treated Jurkat cell lysates (CytC) from a control extract kit (Cell Signaling, #9663) were used as positive controls

To rule out that the observed decrease in cell number during EV collection phase (day 10 to 13) in 3D culture as shown in Fig. 5B is a result of senescence or apoptosis, lysates of HFP-MSCs harvested after 3D culture were probed for expression of p21 and caspase 3 cleavage, respectively (Fig. 5). An aliquot of HFP-MSCs taken before 3D culture served as control (CTRL). As positive controls, cells were treated in 2D with 5 µM etoposide for 48 h or with 30 µM H_2_O_2_ (corresponding to approximately 0.001% (w/v)) for 2 h to induce senescence and p21 expression via oxidative stress. Panel A in Fig. 5 clearly shows that p21 expression after 3D culture at EV harvest is at or below CTRL, while the positive controls display elevated p21 expression as expected. Panel B in Fig. 5 highlights evidently that 3D culture does not induce apoptosis as indicated not only by absent cleavage of pro-caspase 3, but even decreased expression of pro-caspase 3 in presence of either FCS, CPRP or hypACT. Interestingly, lysate of cells treated with H_2_O_2_ also resulted in a similar reduction of caspase 3 expression, however, this sample was loaded simply out of curiosity without intending to induce apoptosis via H_2_O_2_. The employed H_2_O_2_ concentration and exposure time was probably to low to induce apoptosis [39]. Nevertheless, the postive control (CytC) containing cleaved caspase 3 as a result of cytochrome C treated Jurkat cell extract (Cell Signaling, #9663) clearly shows cleaved caspase 3, which is not present in the HFP-MSC lysates neither before or after bioreactor culture. Taken together, the data indicate that neither apoptosis is triggered during 3D culture nor senescence is induced, which would potentially lead to contamination of EV preparations with apoptotic bodies or senescence-associated factors.

### EV enrichment via UF

**Fig. 6:**
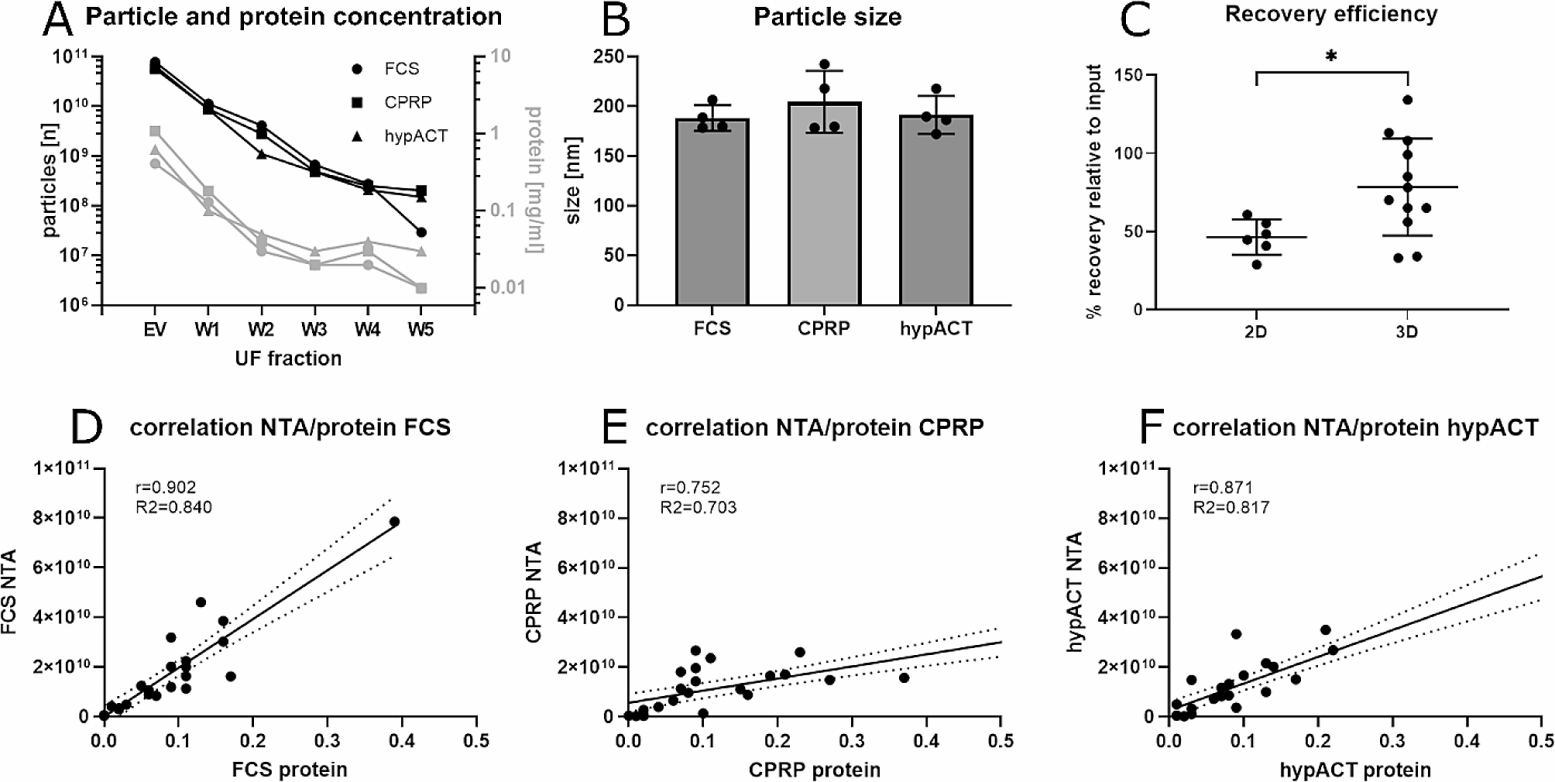
Characterisation of particle size and concentration as well as particle recovery efficiency and correlation with protein amount via NTA after enrichment via ultrafiltration. (A) Analysis of particle and protein concentration in fractions after ultrafiltration (UF) from a representative batch of 3 bioreactors operated in parallel under different treatment conditions (FCS, CPRP, hypACT). (B) Particle size of EV preparations from 4 donors in each of the indicated treatment conditions. Data are given as mean ± SD. (C) Recovery efficiency of particle enrichment via UF comparing amount of particles in input conditioned supernatant and amount of particles in final EV preparation. Data are given as mean ± SD. * . *p* < 0.05. (D-F) Correlation analysis of particle and protein concentrations in UF fractions (EV, W1-W5) from 4 donors

Conditioned media were processed via ultrafiltration for EV enrichment. After processing the whole volume of a given sample, a primary retentate (“EV” fraction) was recovered from the filter units. To quantitatively recover particles from the filter and to detach adsorbed particles, five consecutive clean outs were performed (fraction “W1” to “W5”) using PBS as eluent (Fig. 6A). Neither the total number of recovered particles (F(2,9) = 1.129; *p* = 0.3651, data not shown), nor the total amount of recovered protein (F(2,9) = 1.131; *p* = 3645, data not shown) or mode sizes of the recovered particles (F(2,9) = 0.5868; *p* = 0.576, Fig. 6B) differed between the treatment conditions FCS, CPRP or hypACT of 3D bioreactor cultures. Interestingly, the recovery efficiency differed significantly when comparing 3D bioreactor cultures to 2D flask cultures across all conditions (t(16) = 2.408, *p* = 0.0285) (Fig. 6C). To estimate whether protein concentration is a predictor of particle concentration, correlation and linear regression analyses were performed (Fig. 6D-F). Spearman correlation coefficients were determined as the data were not normally distributed. As a result, particle concentrations correlated with measured protein concentrations significantly for EV preparations from FCS (*r* = 0.902, *p* < 0.0001), CPRP (*r* = 0.752, *p* < 0.0001) and hypACT (*r* = 0.871, *p* < 0.0001) treated 3D bioreactor cultures. To confirm that the HFP-MSCs retained their stemness after bioreactor culture and EV production, cells were detached from the microcarriers and subjected to flowcytometric characterisation of MSC stemness markers CD90, CD73 and CD105. As presented in supplementary Fig. 2, CD90, CD73 and CD105 were clearly detected indicating that the cells retained their stemness.

**Fig. 7:**
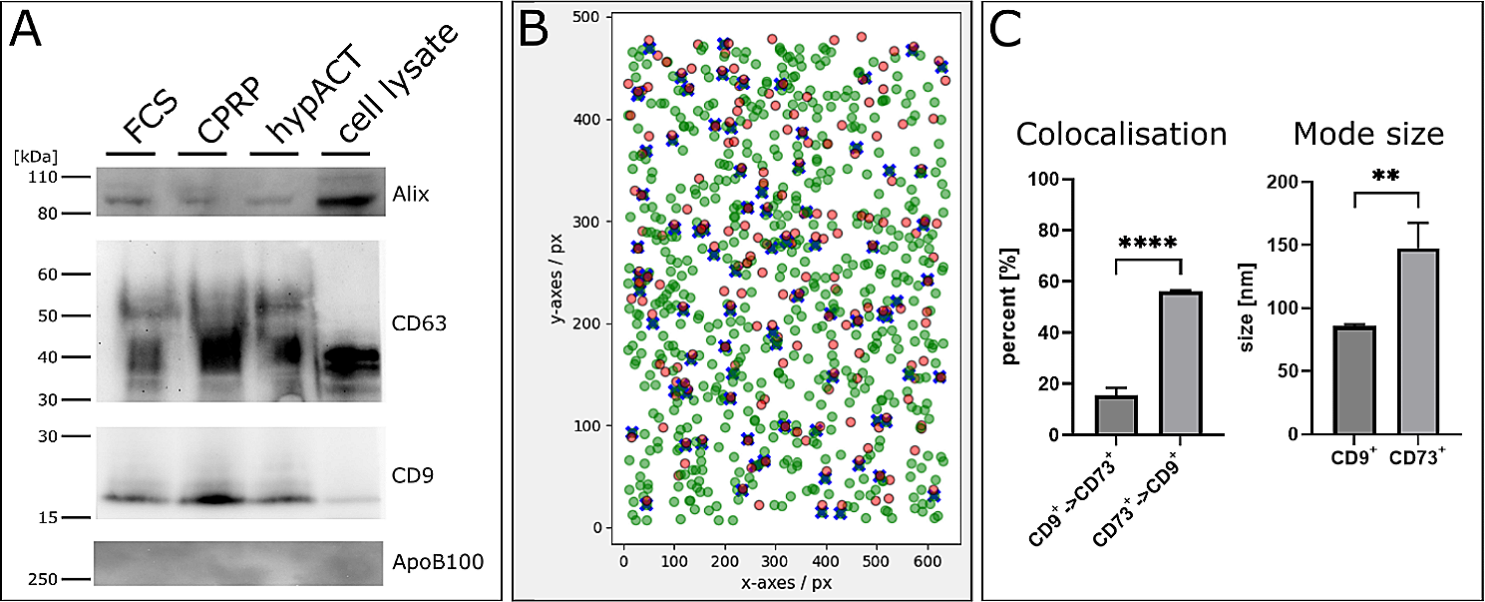
Characterisation of EV marker protein presence via Western Blot and EV marker protein colocalisation via fluorescence NTA. (A) Detection of EV markers CD9, CD63 and Alix as well as the negative marker ApoB100 indicating presence of EVs and absence of contaminants from blood product treatments via Western Blot. (B) Representative result of colocalisation of CD9^+^ (green) and CD73^+^ (red) particles via fluorescence NTA. Blue crosses indicate colocalisation. (C) Left, quantitative assessment of CD9^+^ EVs colocalising with CD73 and CD73^+^ EVs colocalising with CD9 as determined via NTA in fluorescence mode. Right, comparison of mode size of CD9^+^ and CD73^+^ single positive EVs. Data are given as mean ± SD of a representative donor. **** . *p* < 0.0001, ** . *p* < 0.01

To confirm the presence of EVs in the particle preparations, EV marker proteins CD9, CD63 and Alix were detected via Western Blot (Fig. 7). While the EV marker proteins were successfully found in EV preparations with HFP-MSC cell lysate as control, the absence of apolipoprotein B100 indicates that no contaminants from growth medium or the blood product treatment were carried over into the EV preparations. The result was further corroborated via colocalisation of CD9 with CD73 on individual EVs using fluorescence NTA to show not only the cell origin of the enriched particles, but also to qualify the EV character. The ratios of colocalisation differed significantly (t(10) = 33.45, *p* < 0.0001), 15.5 ± 2.92% (SD) of CD9^+^ EVs were also positive for CD73 and 56.0 ± 0.52% (SD) of CD73^+^ particles were also positive for CD9 (Fig. 7C). Surprisingly, the mode size of either CD9^+^ or CD73^+^ particles differed significantly (t(4) = 5.297, *p* = 0.0061), indicating that CD73 was preferentially present on larger EVs (147.5 ± 20.13 nm (SD)), while smaller EVs tended to be characterised by CD9 (85.75 ± 1.552 nm (SD)) across all treatment conditions.

### Comparison of EV yield from 2D and 3D culture

Particle size, particle concentration or protein concentration were not significantly different between conditions (FCS, CPRP, hypACT) for EV preparations from either 2D or 3D culture as determined via NTA and DC assay. Pairwise comparisons of treatment conditions via one-way ANOVA and Dunett posthoc test reached only for the parameters protein amount per EV and number of EV per µg protein marginal significance for EVs derived from CPRP-treated 3D cultures (*p* = 0.0278 and *p* = 0.0461, respectively). This indicated that the treatment of cells with CPRP or hypACT prior to EV collection compared to FCS did not substantially influence the quantitative characteristics of EV yield within 2D or 3D culture groups. However, comparing EV yield between 2D and 3D cultures revealed substantial differences.

**Fig. 8:**
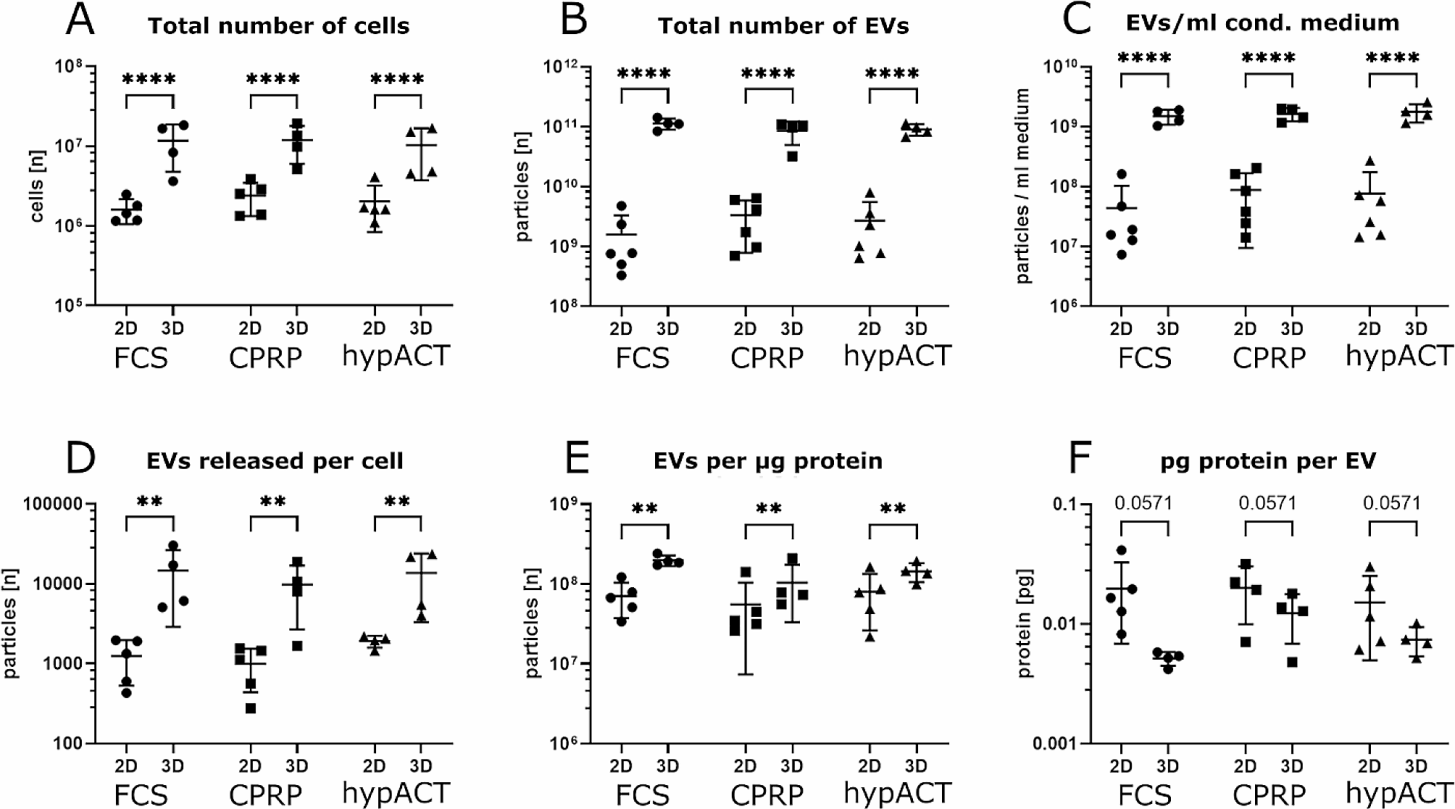
Comparison of EV yield from HFP-MSCs in 2D and 3D vertical wheel bioreactor culture. Data are given as mean ± SD from up to 6 EV production batches involving up to 5 individual donors. Statistical significance was determined via two-way ANOVA and Tukey posthoc test. ** . *p* < 0.005, **** . *p* < 0.0001

As the 3D bioreactor involved a much larger culture surface available for cell attachment due to the use of microcarriers (175 cm^2^ vs. 576 cm^2^ per batch), the total cell number per batch was significantly higher with 1.13*10^7^±7.51*10^6^ (SD) cells in 3D bioreactors compared to 2.02*10^6^±1.1*10^6^ (SD) cells in 2D flasks (Fig. 8A; F(1,23) = 33.60; *p* < 0.0001). Although the available culture surface was larger in 3D by a factor of 3.29, the total number of cells at harvest increased by a factor of 5.77 ± 1.31 (SD). The number of total EVs harvested per batch from 2D and 3D cultures showed a log difference of roughly 2 irrespective of the treatment condition with exact fold change of 43.47 ± 24.58 (SD) on average from 3D compared to 2D (Fig. 8B; F(1,26) = 206.9; *p* < 0.0001). Accordingly, the number of particles per ml conditioned medium before EV isolation differed significantly between 2D and 3D (Fig. 8C; F(1,26) = 201.1; *p* < 0.0001). Interestingly, also the number of EVs released per cell was significantly higher in 3D culture (Fig. 8D; F(1,22) = 19.79; *p* = 0.0002). As the protein amount correlated with the number of EVs obtained via ultrafiltration (Fig. 6), the EV:protein ratio was compared between 2D and 3D cultures resulting in significantly higher number of EVs per µg of protein (Fig. 8E; F(1,23) = 17.62; *p* = 0.0003). In contrast, the absolute amount of protein per individual EV was lower in 3D HFP-MSC-EVs, although missing statistical significance (Fig. 8F).

### Comparison of biological activity of EVs from 2D and 3D culture

**Fig. 9:**
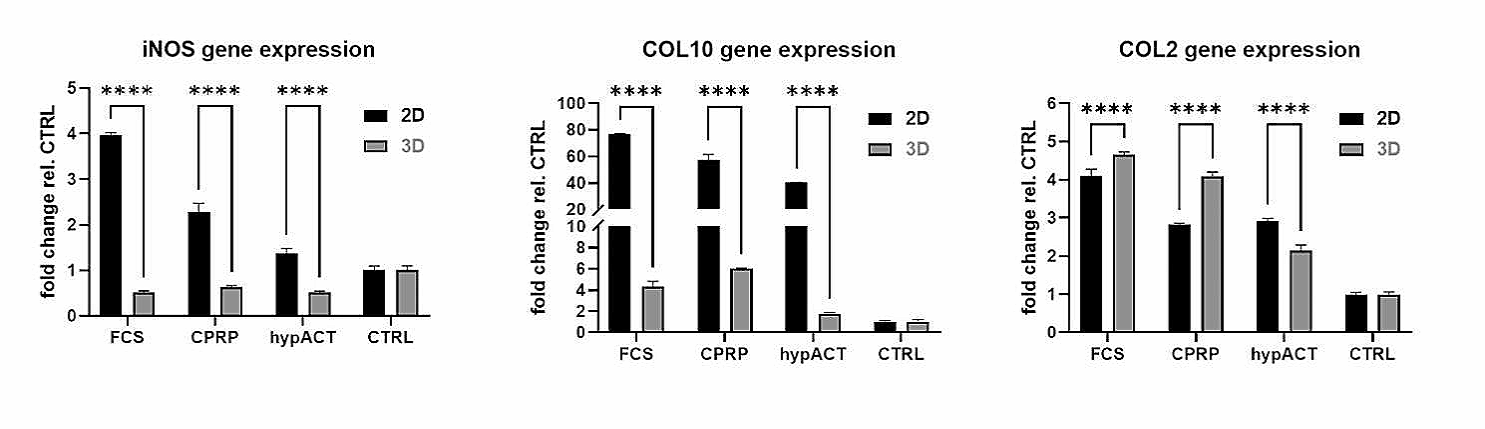
Comparison of potency of HFP-MSC-EVs from 2D and 3D culture. Data are given as mean ± SD from one chondrocyte donor with three technical replicates. Statistical significance was determined via two-way ANOVA and Tukey posthoc test. **** . *p* < 0.0001

To assess the potency of HFP-MSC-EVs generated in vertical wheel bioreactor culture (3D EVs), gene expression analysis of iNOS, COL10 and COL2 in OA chondrocytes from one patient treated with 50,000 EVs per cell was performed via RT-qPCR in comparison to EVs obtained from HFP-MSCs cultured in flasks (2D EVs). HFP-MSCs from the same donor were used to generate 2D and 3D EVs for this experiment. iNOS expression was significantly enhanced by 2D EVs from FCS-, CPRP- or hypACT-supplemented culture compared to untreated chondrocytes (CTRL). 3D EVs decreased iNOS expression compared to CTRL (Fig. 9). Although 3D EVs elevated collagen type X (COL10) expression compared to CTRL, COL10 expression in response to 2D EVs was substantially increased compared to 3D EVs. Collagen type II (COL2) expression was elevated in all treatment conditions compared to untreated cells. Nevertheless, 3D HFP-MSC-EVs from FCS- and CPRP-supplemented culture induced higher COL2 expression than 2D EVs. Taken together, the potency of HFP-MSC-EVs from vertical wheel bioreactor microcarrier suspension culture shows promising effects with respect to inhibition of inflammation, inhibition of chondrocyte hypertrophy and cartilage-specific extracellular matrix production compared to EVs generated via conventional 2D culture.

## Discussion

This study explored the use of HFP-MSCs as cell source for production of EVs. Standard 2D culture of HFP-MSCs was compared to a 3D microcarrier-based suspension culture system. To simulate an intra-articular injection therapy increasingly applied in orthopaedic care, cells were exposed to human blood products CPRP and hypACT with FCS as control with the intention to stimulate an EV release from HFP-MSCs that encodes a response to this treatment.

Directly after HFP-MSC isolation from tissues, metabolic activity was followed over 14 days (Fig. 2). The results indicate that human-derived media supplements could replace animal-based sera for the culture of HFP-MSCs and maybe production of HFP-MSC-derived EVs to establish an EV production process free from animal components. However, this will require replacement of FCS during expansion phase of the bioreactor culture, which was not yet done in the study, because a treatment phase was included in the EV production procedure. This approach might also allow shortening of the expansion phase as cells were metabolically more active in presence of CPRP or hypACT (Fig. 2).

The trilineage differentiation potential of the isolated cells was successfully determined. Together with their adherence capacity and the expression of a consensus of stemness markers (Figs. 2 and 3), these characteristics qualify the cells as MSCs [9]. Nevertheless, the differentiation capacities of MSCs from individual donors in response to different blood products might vary as indicated by the low adipogenic differentiation potential in presence of hypACT, because this observation is contrary to results from an earlier study in our group where hypACT promoted adipogenic differentiation most strongly [20].

The flowcytometric data on MSC stemness marker expression after isolation showed a relatively low signal intensity for CD105 compared to other markers (CD90, CD73) (Fig. 2). At a first glance, this gives the idea about low CD105 expression in HFP-MSCs, however, a quantitative statement cannot be made from the data, as the antibodies are conjugated to different fluorophors and conjugation ratio as well as quantum yields of the fluorophores might differ, and thus signal strength. Nevertheless, CD105-negative MSCs were defined as specific subpopulation of MSCs with distinct differentiation and immunomodulatory capacities in mice [3]. CD105-negative MSCs from adipose and umbilical cord tissue were also found having stronger T-cell regulatory function and releasing lower amounts of TGFβ1 than CD105-positive MSCs [31]. CD105^high^ adipose MSCs have marked adipogenic potential, while CD105^low^ adipose MSCs displayed improved osteogenesis again associated with decreased TGF-β1 signaling [23].

Production of MSC EVs for therapeutic applications is a growing field also in the context of bone and cartilage diseases [14, 15, 19, 22, 27]. Different 3D culture approaches are investigated including stirred tank bioreactors [32] and vertical wheel bioreactors [6], however, there are still standardisations to be developed and problems to be solved. One of them is the decrease in cell number during EV collection observed in this study (Fig. 4) and elsewhere [6]. EV collection media are often serum free to avoid contamination of MSC EV preparations with bovine FCS EVs, for example. Although there are EV-depleted sera available, the use of animal components for therapeutic EV production should be avoided for ethical reasons as well as for inherent heterogeneity of the composition from batch to batch. Last but not least, avoiding the use of animal products prevents transmission of diseases. Nevetheless, the question remains how to stabilize cell number during EV collection. We could rule out that apoptosis underlies this phenomenon (Fig. 5). Possibly, the use of lipid supplementation to the serum free medium could help as EV release uses up lipid membrane available to cells and energy, which might be missing for *de novo* biosynthesis of lipids.

Ultrafiltration was the method of choice to enrich EVs from conditioned supernatant instead of ultracentrifugation or size-exclusion chromatography [26] as EV collection media were serum free and relatively large volumes were processed. The EV enrichment method might influence the proportion of co-isolates such as soluble total protein, which tends to form aggregates during ultracentrifugation or precipitation-based enrichment methods [1] and therefore total protein concentration cannot be used to describe the concentration of EVs. This circumstance takes on special significance when it comes to dosing of EVs. Apparently, using the described protocol (Fig. 6) involving ultrafiltration allows using total protein concentration to estimate EV concentration. Interestingly, there was a substantial difference in the recovery efficiency of particles from conditioned supernatant from 2D and 3D culture. As the total number of particles was lower in 2D culture, a higher proportion could have been lost during enrichment by non-specifically attaching to the filtration unit.

The characterisation of EVs from 3D culture via NTA in fluorescence mode revealed a co-localisation of CD9-positive EVs with CD73 (Fig. 7). However, the mode sizes differed having CD9 on smaller particles and CD73 on larger particles. This could be explained via a preferential biosynthesis of CD9-positive EVs at intracellular membranes resulting in the formation of exosomes, while larger CD73-positive particles could derive from the plasma membrane where CD73 is localised in MSCs [17]. However, their co-localisation underlines the difficulty of using one or the other marker to describe a specific type of EV [35].

The observed increase in EV yield from 2D to 3D culture was not only based on higher number of cultured cells due to larger surface area available for attachment, but also a higher number of EVs released per cell (Fig. 8). While approximately 50-fold more EVs on average were obtained in 3D culture of HFP-MSCs in this study, EV yield increased up to 9.4-fold in 3D culture of umbilical cord MSCs [18] and up to 100-fold more EVs were released from endothelial cells [28]. An explanation for these phenomena could be mechanical stimulation experienced by cells in dynamic 3D culture [8] that could not only stimulate EV release, but also prevent or reduce the reuptake rate of EVs by neighbouring cells. Besides the choice of the culture system and associated different mechanical stimulation, other factors such as the availability of oxygen could influence EVs released from MSCs. EV production under hypoxia not only increased EV yield, but also modulated the cargo profile and increased the pro-angiogenic activity [2, 12]. The vast increase in EV yield from 2D to 3D in this study could possibly still be an underestimation of particle concentration in NTA, as the sensitivity settings were chosen to limit background signals. This might have led to miss signals from some small EVs.

A limitation of the study is the fact that HFP-MSCs were isolated from OA patients. Comparing HFP-MSC-EVs isolated from patient cells and healthy donor cells could be worthwile to study in future pre-clinical evaluations. Nevertheless, a potency assay assessing biological activity of HFP-MSC-EVs from 3D VWBR culture in comparison to 2D culture revealed decreased iNOS expression, lower COL10 expression and favoured COL2 expression which could be beneficial outcomes for OA patients and could contribute to OA disease modifying effects. In addition, the presented protocol needs adaptions to ensure a GMP-compliant EV production procedure. For example, isolation of primary HFP-MSC was performed in presence of FCS-supplemented culture media and which should be replaced by xeno-free media ideally also chemically-defined. Similarly, ultrafiltration columns were saturated with BSA during EV isolation. To implement the protocol for clinical grade EV production, the use of BSA should be avoided in favour of human albumin. Similarly, the use of bovine or human blood products during culture could lead to trace amounts of contaminants in the final EV preparations. However, the absence of apolipoprotein B100, which is indicative of low density lipoprotein particles that have similar size as EVs, suggests that potential particular contaminants from blood products carried over into the EV preparation are very low as shown in Fig. 7A. Nevertheless, the investigated EV production procedure holds promise to generate substantially more EVs under probably physiologically more relevant conditions compared to 2D culture. Additionally, a potency evaluation indicated that the 3D culture approach yields EVs with favourable biologic activity as outlined by the gene expression changes induced in OA chondrocytes (Fig. 9).

## Conclusions

The presented protocol and its characterisation to produce HFP-MSC-derived EVs in a vertical wheel 3D suspension bioreactor resulted in the generation of sufficient amounts of EVs to perform further studies for pre-clinical evaluation and dose response experiments. In addition, the comparison to 2D culture highlights the optimisation regarding media consumption per EV yield to implement a sustainable EV production procedure. In addition to quantitative improvements, qualitative characteristics of 3D generated EVs favour inhibition of inflammation, reduced chondrocyte hypertrophy and promotion of cartilage-specific ECM production.

### Electronic supplementary material

Below is the link to the electronic supplementary material.

Supplementary Material 1

Supplementary Material 2

## Data Availability

All data generated or analysed during this study are included in this published article.

## Abbreviations

CPRP: citrate anticoagulated platelet–rich plasma
DMEM: dulbecco’s modified eagle medium
EV: extracellular vesicle
FCS: fetal calf serum
HFP: hoffa fat pad
hypACT: hyperacute serum
MSC: mesenchymal stem cell
NTA: nanoparticle tracking analysis
OA: osteoarthritis
UF: ultrafiltration
VWBR: vertical whee bioreactor
WB: Western Blot
BSA: Bovine serum albumin
