# Supplementary material for: Culture of Hoffa fat pad mesenchymal stem/stromal cells on microcarrier suspension in vertical wheel bioreactor for extracellular vesicle production
Source: Stem Cell Res Ther. 2024 Mar 5;15:61. doi: 10.1186/s13287-024-03681-9 (PMC10913578; doi:10.1186/s13287-024-03681-9)
Supplement: Supplementary file 1 — Supplementary Material 1 [file 13287_2024_3681_MOESM1_ESM.docx]

# Supplementary information – Uncropped blot images

## Apoptosis and senescence marker characterisation

The blots below were used to analyze expression of cleaved caspase 3 and p21 in HFP-MSCs after EV harvest.


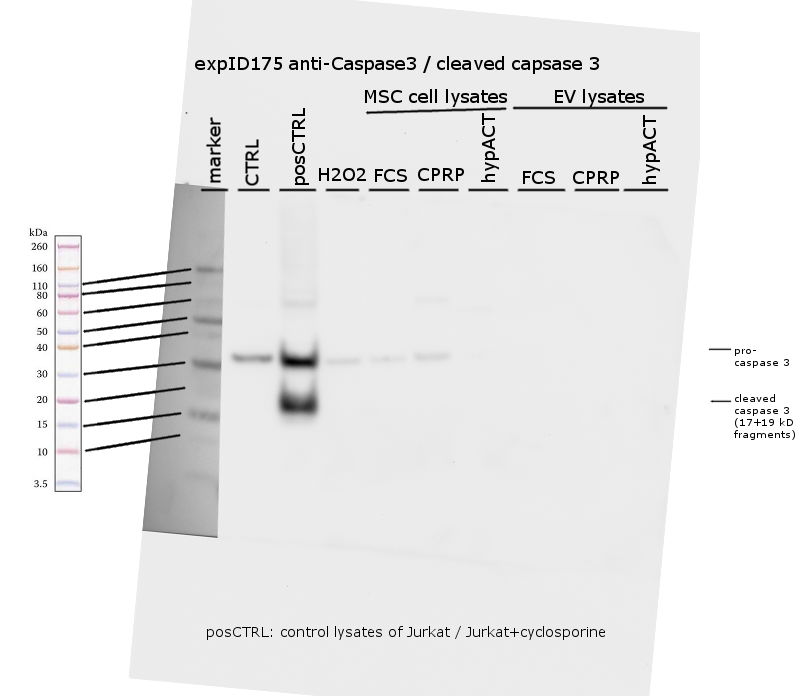


Figure S1. Detection of cleaved caspase in 10 µg of lysed HFP-MSCs and HFP-MSC-EVs on Western Blot. Cropped image shown in Figure 5.


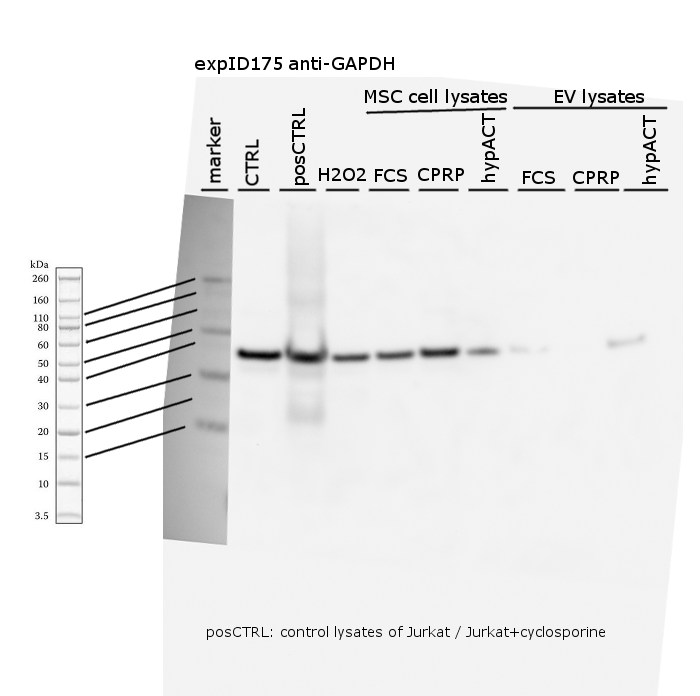


Figure S2. Detection of GAPDH in 10 µg of lysed HFP-MSCs and HFP-MSC-EVs on Western Blot, reprobed membrane from Figure S1. Cropped image shown in Figure 5.


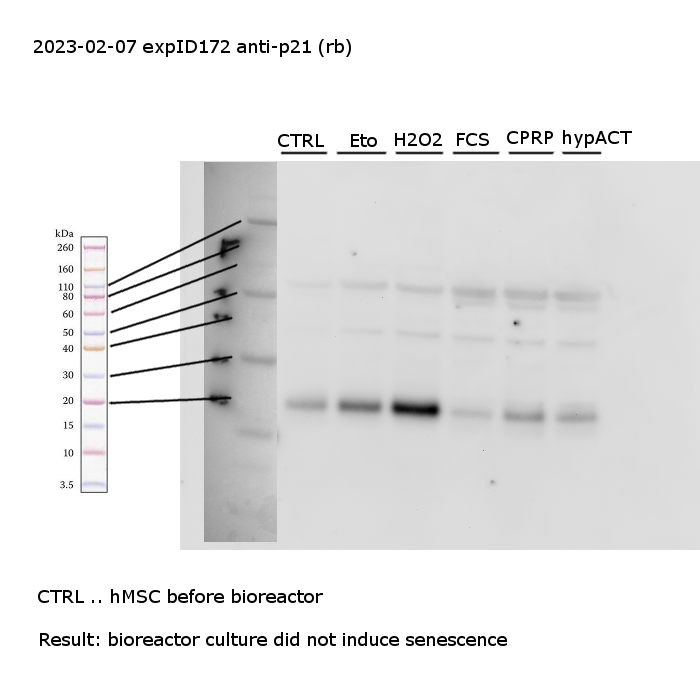


Figure S3. Detection of p21 in 10 µg of lysed HFP-MSCs treated with the indicated blood products on Western Blot. Cropped image shown in Figure 5.


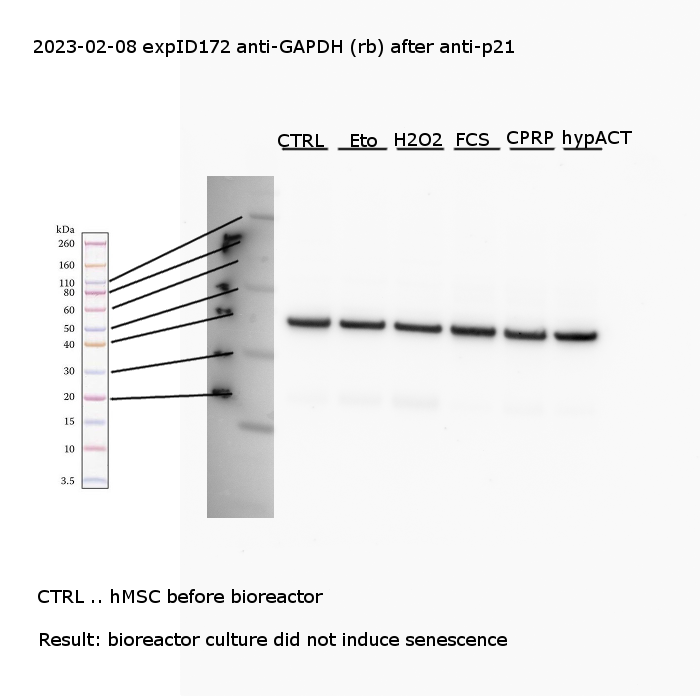


Figure S4. Detection of GAPDH in 10 µg of lysed HFP-MSCs treated with the indicated blood products on Western Blot, reprobed membrane from Figure S1. Cropped image shown in Figure 5.

## EV protein marker characterisation

The blots below were used to show presence of EV marker proteins (CD9, CD63, Alix) or absence of contaminating material (ApoB100).


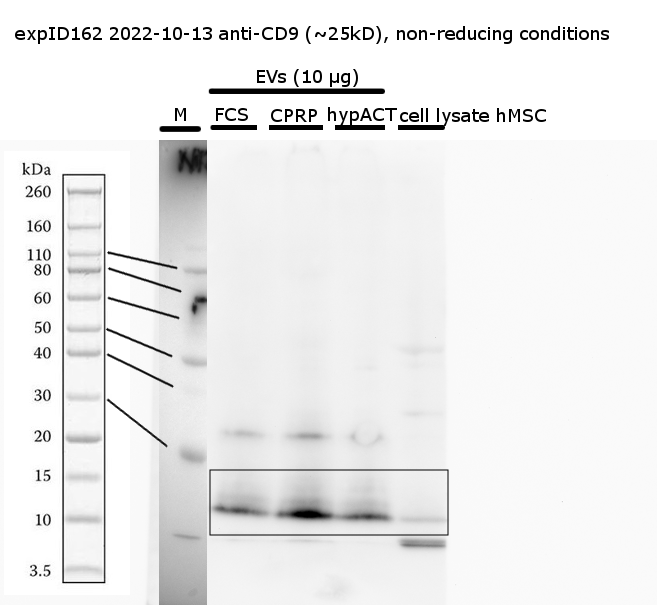


Figure S5. Detection of CD9 in 10 µg of lysed EVs compared to HFP-MSC cell lysate on Western Blot. The box outlines the cropped image used in Figure 7.


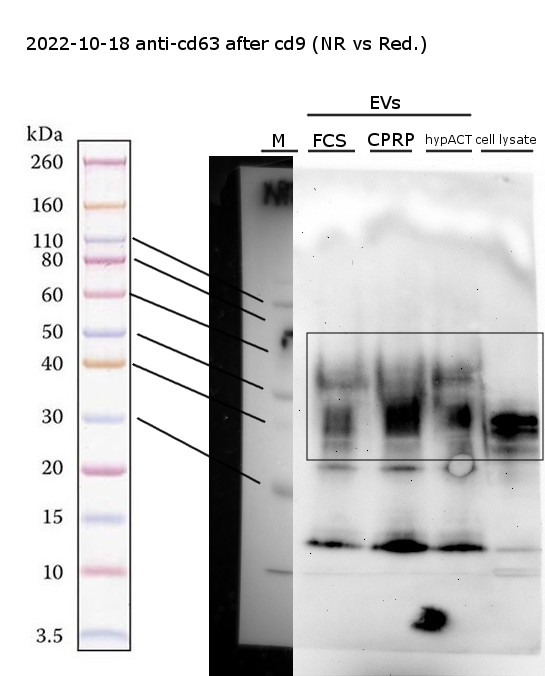


Figure S6. Detection of CD63 after CD9 on the same membrane in 10 µg of lysed EVs compared to HFP-MSC cell lysate on Western Blot. The box outlines the cropped image used in Figure 7.


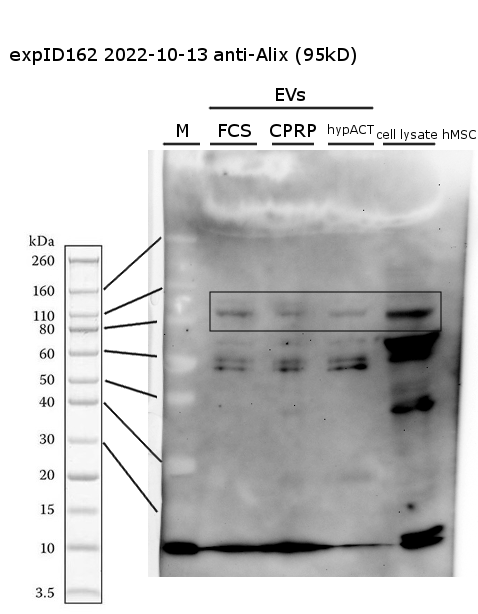


Figure S7. Detection of Alix in 10 µg of lysed EVs compared to HFP-MSC cell lysate on Western Blot. The box outlines the cropped image used in Figure 7.


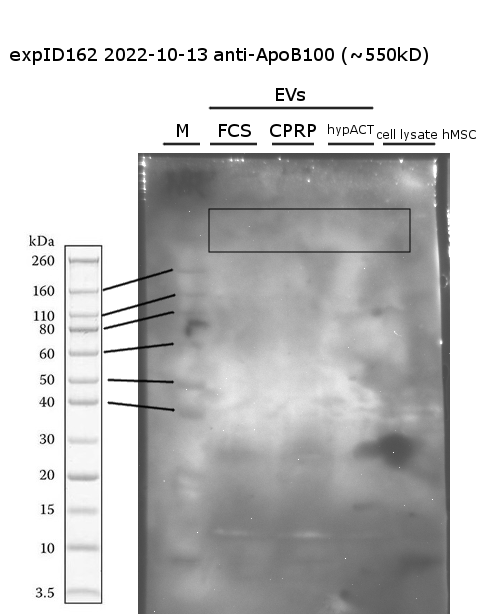


Figure S8. Detection of ApoB100 in 10 µg of lysed EVs compared to HFP-MSC cell lysate on Western Blot. The box outlines the cropped image used in Figure 7.
