# Supplementary material for: Culture of Hoffa fat pad mesenchymal stem/stromal cells on microcarrier suspension in vertical wheel bioreactor for extracellular vesicle production
Source: Stem Cell Res Ther. 2024 Mar 5;15:61. doi: 10.1186/s13287-024-03681-9 (PMC10913578; doi:10.1186/s13287-024-03681-9)
Supplement: Supplementary file 2 — Supplementary Material 2 [file 13287_2024_3681_MOESM2_ESM.docx]

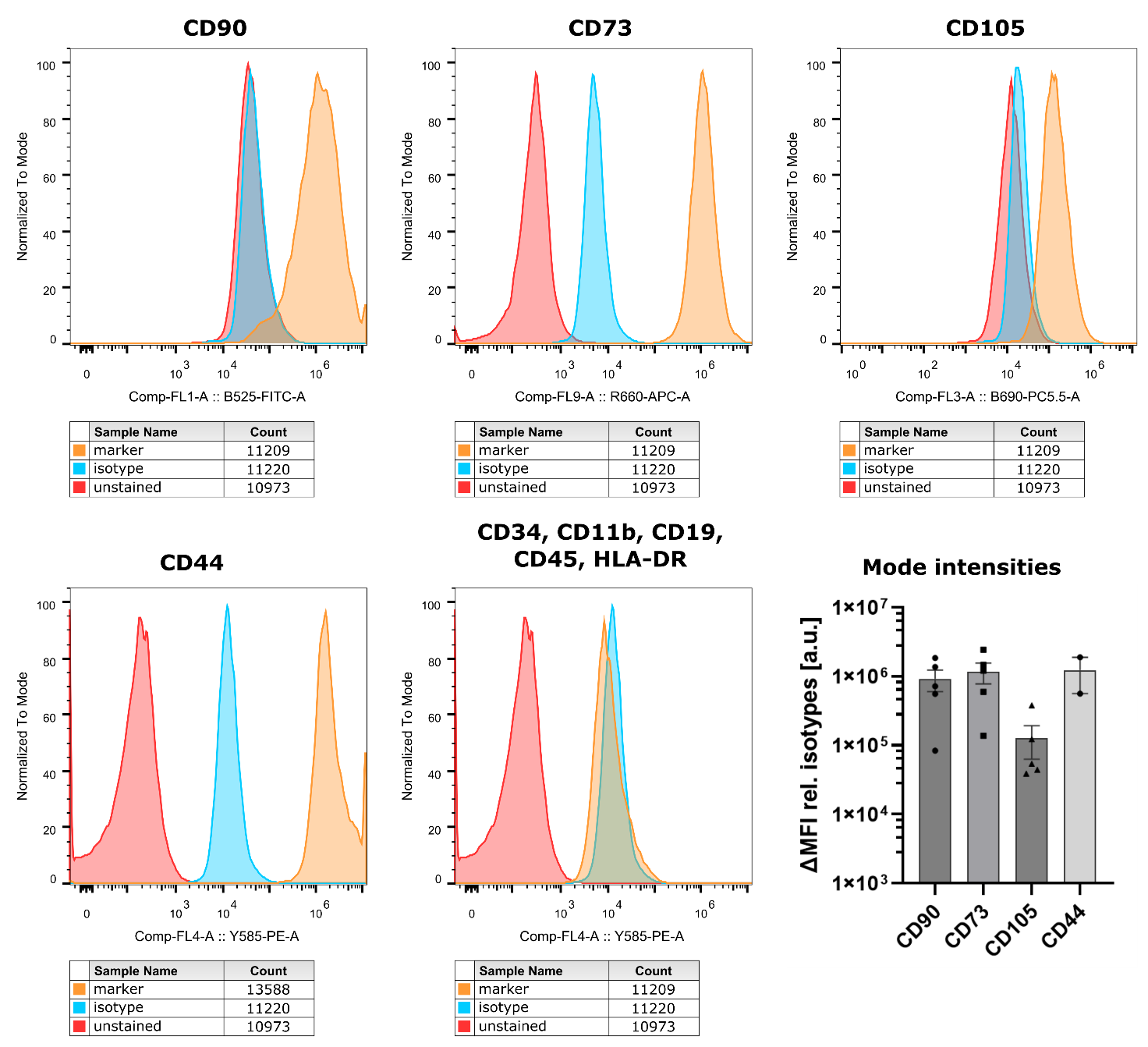


Supplementary figure 1. Flow cytometric characterization of isolated HFP-MSCs after adherence. Histograms from a representative donor are shown, the bar plot summarizes mode fluorescence intensity of the indicated markers for up to n=5 donors. Data are given as mean ± SEM.


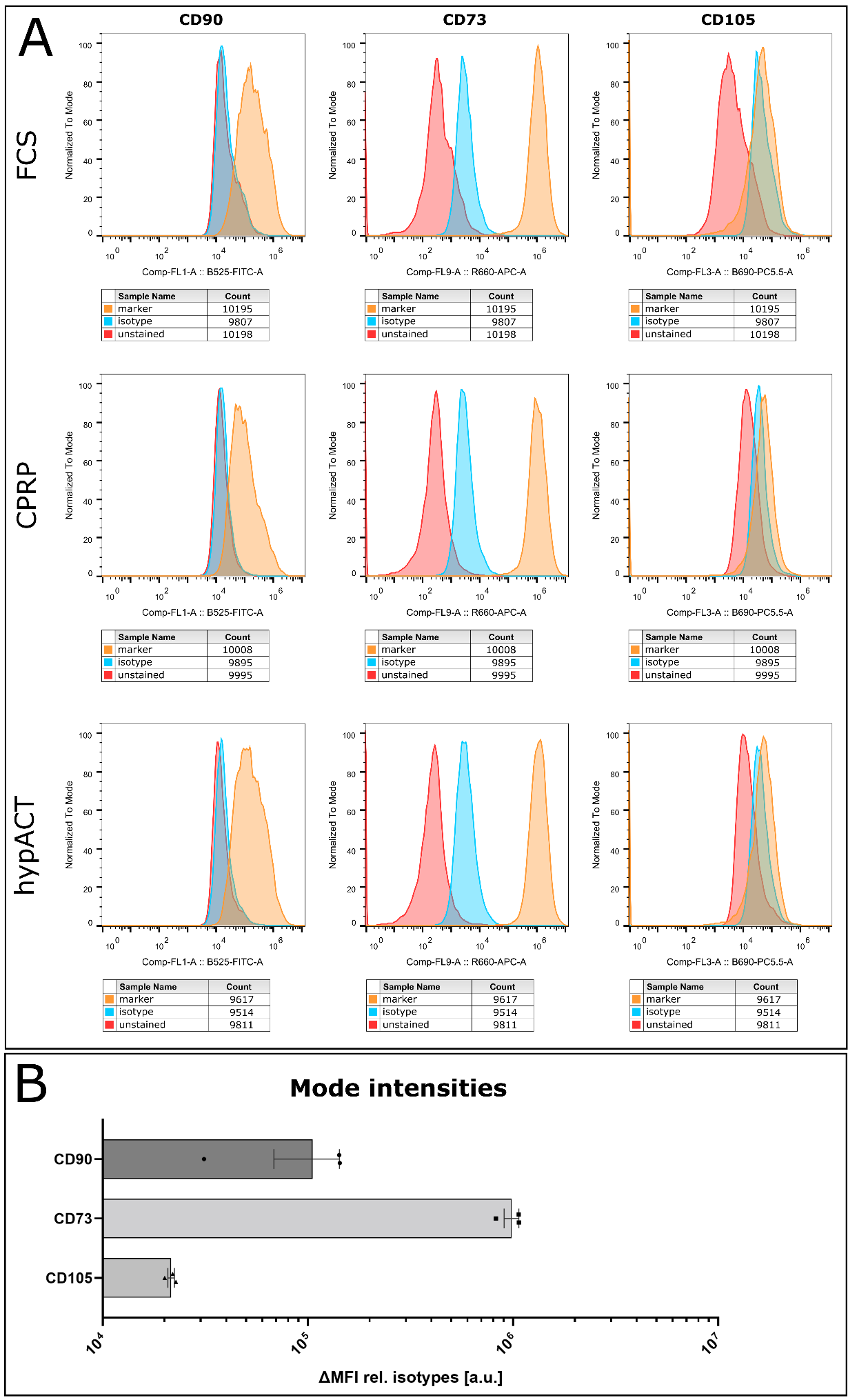


Supplementary figure 2. Flow cytometric characterization of HFP-MSCs after EV production. (A) Histograms from a representative batch of three bioreactors are shown operated with cells treated with either FCS, CPRP or hypACT from a single donor; (B) mode fluorescence intensity of the indicated markers of the batch of bioreactors presented in panel A. Data are given as mean ± SEM.

| Gene | Forward | reverse |
| --- | --- | --- |
| iNOS | gaccagtacgtttggcaatg | tttcagcatgaagagcgattt |
| COL10 | caaggcaccatctccagg | tgggcatttggtatcgttcag |
| COL2 | gtgtcagggccaggatgt | tcccagtgtcacagacacagat |
| GAPDH | ctctgctcctcctgttcgac | acgaccaaatccgttgactc |

Table 1. List of primers used for RT-qPCR analyses.
